# Incomplete silencing of full mutation alleles in males with fragile X syndrome is associated with autistic features

**DOI:** 10.1186/s13229-019-0271-7

**Published:** 2019-05-03

**Authors:** Emma K. Baker, Marta Arpone, Solange M. Aliaga, Lesley Bretherton, Claudine M. Kraan, Minh Bui, Howard R. Slater, Ling Ling, David Francis, Matthew F. Hunter, Justine Elliott, Carolyn Rogers, Michael Field, Jonathan Cohen, Kim Cornish, Lorena Santa Maria, Victor Faundes, Bianca Curotto, Paulina Morales, Cesar Trigo, Isabel Salas, Angelica M. Alliende, David J. Amor, David E. Godler

**Affiliations:** 1 Diagnosis and Development, Murdoch Children’s Research Institute, Royal Children’s Hospital, 50 Flemington Rd, Parkville, VIC 3052 Australia; 2 Department of Paediatrics, Faculty of Medicine, Dentistry and Health Sciences, University of Melbourne, Parkville, Australia; 3 Brain and Mind, Murdoch Children’s Research Institute, Royal Children’s Hospital, Melbourne, Australia; 4 Centre for Epidemiology and Biostatistics, Melbourne School of Population and Global Health, University of Melbourne, Carlton, Australia; 5 Victorian Clinical Genetics Services and Murdoch Children’s Research Institute, Royal Children’s Hospital, Melbourne, VIC Australia; 6 Monash Genetics, Monash Health, Melbourne, VIC Australia; 7 Department of Paediatrics, Monash University, Clayton, VIC Australia; 8 Genetics of Learning Disability Service, Hunter Genetics, Waratah, NSW Australia; 9 Fragile X Alliance Inc, North Caulfield, VIC and Center for Developmental Disability Health Victoria, Monash University, Clayton, Australia; 10 Monash Institute of Cognitive and Clinical Neurosciences, Monash University, Clayton, VIC Australia; 11 Molecular and Cytogenetics Laboratory, INTA, University of Chile, Santiago, Chile; 12 Neurodisability and Rehabilitation, Murdoch Children’s Research Institute, Royal Children’s Hospital, Melbourne, Australia

**Keywords:** Fragile X syndrome, *FMR1* mRNA, Autism, Intellectual disability, Mosaicism

## Abstract

**Background:**

Fragile X syndrome (FXS) is a common monogenic cause of intellectual disability with autism features. While it is caused by loss of the *FMR*1 product (FMRP), mosaicism for active and inactive *FMR1* alleles, including alleles termed premutation (PM: 55–199 CGGs), is not uncommon. Importantly, both PM and active full mutation (FM: ≥ 200 CGGs) alleles often express elevated levels of mRNA that are thought to be toxic. This study determined if complete *FMR1* mRNA silencing from FM alleles and/or levels of *FMR1* mRNA (if present) in blood are associated with intellectual functioning and autism features in FXS.

**Methods:**

The study cohort included 98 participants (70.4% male) with FXS (FM-only and PM/FM mosaic) aged 1–43 years. A control group of 14 females were used to establish control *FMR1* mRNA reference range. Intellectual functioning and autism features were assessed using the Mullen Scales of Early Learning or an age-appropriate Wechsler Scale and the Autism Diagnostic Observation Schedule-2nd Edition (ADOS-2), respectively. *FMR1* mRNA was analysed in venous blood collected at the time of assessments, using the real-time PCR relative standard curve method.

**Results:**

Females with FXS had significantly higher levels of *FMR1* mRNA (*p* < 0.001) than males. *FMR1* mRNA levels were positively associated with age (*p* < 0.001), but not with intellectual functioning and autistic features in females. FM-only males (aged < 19 years) expressing FM *FMR1* mRNA had significantly higher ADOS calibrated severity scores compared to FM-only males with completely silenced *FMR1* (*p* = 0.011). However, there were no significant differences between these subgroups on intellectual functioning. In contrast, decreased levels of *FMR1* mRNA were associated with decreased intellectual functioning in FXS males (*p* = 0.029), but not autism features, when combined with the PM/FM mosaic group.

**Conclusion:**

Incomplete silencing of toxic FM RNA may be associated with autistic features, but not intellectual functioning in FXS males. While decreased levels of mRNA may be more predictive of intellectual functioning than autism features. If confirmed in future studies, these findings may have implications for patient stratification, outcome measure development, and design of clinical and pre-clinical trials in FXS.

**Electronic supplementary material:**

The online version of this article (10.1186/s13229-019-0271-7) contains supplementary material, which is available to authorized users.

## Background

Fragile X Syndrome (FXS) is a common monogenic syndrome associated with intellectual disability (ID) and autism features, caused by a trinucleotide CGG expansion (≥ 200 repeats), termed full mutation (FM) [1]. FM alleles are usually associated with increased methylation of the *FMR1* promoter extending into *FMR1* intron 1, and decreased transcription of *FMR1* and loss of its product, fragile X mental retardation protein (FMRP) (reviewed in [2]). FMRP is essential for normal neurodevelopment, with its loss associated with FXS phenotypes including learning and memory deficits, intellectual functioning, behavioural problems, and autism features [3].

*FMR1* alleles with smaller size CGG expansion (55–199 repeats) have been termed premutation (PM). These PM alleles have been reported to have an unmethylated *FMR1* promoter and abnormally increased levels of *FMR1* mRNA [4, 5]. This increased transcription has been postulated to cause “RNA gain of function” toxicity that has been associated with late onset disorders in a proportion of PM carriers [4, 5]. Other potential pathogenic mechanisms described in PM-related disorders include marginally decreased FMRP, expanded repeat associated non-AUG translation, as well as increased transcription of *ASFMR1*/*FMR4* originating from the same locus as *FMR1*, but in the anti-sense direction (reviewed in [2]).

Approximately 12–41% of males with FXS have been reported to have CGG size and/or methylation mosaicism [6, 7]. CGG size mosaicism occurs when some cells contain alleles of different sizes in the same individual. The most common mosaicism reported in FXS [6, 7] is unmethylated PM and FM alleles in some cells and methylated FM alleles in others, defined in this study as PM/FM mosaicism. Mosaicism can also occur in the absence of PM alleles, where some cells have methylated FM alleles that do not express mRNA and others that have unmethylated and transcribed FM alleles.

The previous prevalence estimates of mosaicism in FXS (12–41%) are likely to be under-estimated, as previous studies have defined mosaicism using methylation-sensitive Southern blot, a technique that cannot detect mosaic alleles if present in less than 20% of cells [8]. Attenuated FXS phenotypes have been described in these cases, though significant variability is still observed [9], and autistic features remain common [10]. Several case reports have also indicated that individuals mosaic for unmethylated PM and FM alleles express *FMR1* mRNA from expanded PM and FM alleles, and have a fragile X-associated tremor/ataxia syndrome (FXTAS) phenotype, based on clinical assessments and MRI features [11–13]; though associations with other FXS characteristics have not been explored in large samples of FXS individuals.

While there is a vast amount of literature that has explored the molecular mechanisms that underpin the specific behavioural phenotype of FXS (FM and PM/FM mosaic), these studies have predominantly focused on CGG sizing, FMRP in blood and DNA methylation analyses using Southern blot (reviewed in [2]). Studies examining the associations between *FMR1* mRNA, level of mosaicism and the behavioural phenotypes in FXS males and females are lacking. Moreover, the real-time PCR method most commonly used in previous studies of *FMR1* mRNA “toxicity” normalised *FMR1* mRNA to β-glucuronidase as a single internal control gene, which is not stably expressed in blood, and has been reported to be itself associated with PM-related phenotypes [14, 15].

This is the first study aimed at determining if phenotypic differences exist between FM males with complete and incomplete *FMR1* mRNA silencing in peripheral blood mononuclear cells (PBMCs). The study also explored relationships between the levels of *FMR1* mRNA (if not completely silenced) in PBMCs and severity of intellectual functioning and autism features using improved methodologies for more accurate quantification of mRNA in *FMR1*related disorders [15, 16]. Moreover, with the advent of calibrated severity scores (CSS) for both the social affect and repetitive and restricted behaviour domains of the Autism Diagnostic Observation Schedule (ADOS) [17], this study used a more detailed approach to assess autism features.

## Methods

### Participants

This study comprised a large international cohort of 98 (70.4% male) individuals with FXS recruited in Australia and Chile. Male participants (*n* = 69; 79.7% FM-only) were aged between 1.89 and 43.17 while female (*n* = 29; 75.9% FM-only) participants were aged between 1.71 and 32.52 years. A control group of 14 females (aged 22 to 54 years) was also included for reference ranges of *FMR1* mRNA levels. These individuals had confirmed normal size alleles (CGG < 45), and were recruited as part of previous studies [18].

All participants had undergone fragile X genetic testing prior to recruitment using CGG PCR sizing and Southern blot analysis, as previously described [19, 20]. Individuals were excluded from the study if they had any other genetic conditions of known clinical significance, if they had any significant medical conditions (e.g. stroke, head trauma) and/or if they had inadequately controlled seizures.

### Sample processing

Five millilitre venous blood samples were collected in EDTA tubes at the time of assessment. PBMC isolation was performed using Ficoll gradient separation, as previously described [21], and RNA extracted using RNeasy kit as per manufacturer’s instructions (Qiagen, Germany).

### *FMR1* mRNA analysis

Complementary DNA (cDNA) strand synthesis was then performed on 10 ng of RNA for each sample using the High Capacity cDNA Reverse Transcription kit (Thermo Fisher scientific, Global). ViiA 7 Real-Time PCR System (Life Technologies, Global) was then used for *FMR1* mRNA analysis using the reverse transcription real-time PCR. The relative standard curve method was utilised for *FMR1* 5′ and 3′ mRNA quantification normalised to mRNA of two internal control genes (*EIF4A2* and *SDHA*), as previously described [14, 15], with mean of three technical replicates used to represent relative *FMR1* mRNA levels for each sample in normalised arbitrary units (a.u.).

### Intellectual functioning

Depending on age and country, participants were assessed with one of the following standardised assessments: the Mullen Scales of Early Learning (MSEL; Australian children < 3 years) [22], the Wechsler Preschool and Primary Scale of Intelligence-3rd Edition (WPPSI-III; children aged 3 years to 6 years, 11 months) [23, 24], the Wechsler Intelligence Scale for Children-4th Edition Australian (WISC-IV; Australian children aged 7 years to 16 years, 11 months) [25]/Wechsler Intelligence Scale for Children-3rd Edition Spanish (WISC-III; Chilean children aged 7 years to 16 years, 11 months) [26] or the Wechsler Adult Intelligence Scale-4th Edition Australian and Chilean editions (WAIS-IV; individuals aged 17+ years) [27, 28]. To address the floor effect that is commonly observed for individuals with FXS on standard intellectual functioning assessments, corrected IQ scores (cFSIQ, cVIQ, cPIQ) were used [29]. A limitation to this method in the current study is that the WISC-III (Chilean version) incorporates working memory (WM) and processing speed (PS) tasks in the verbal and perceptual reasoning indexes, and these types of tasks have previously been shown to be a relative deficit in the cognitive profiles of individuals with FXS [30, 31]. Moreover, while Verbal IQ (VIQ), Performance IQ (PIQ) and Full Scale IQ (FSIQ) (early learning composite as a proxy for FSIQ) scores can be derived from the MSEL [32, 33], the assessment is qualitatively different to that of the Wechsler scales. Therefore, in the supplementary materials, the analyses using (i) standard scores and (ii) corrected scores with participants assessed with the MSEL and WISC-III removed are presented.

### Autism features

The Autism Diagnostic Observation Schedule-2nd Edition (ADOS-2) [34] was used to assess autism features. The ADOS-2 is a semi-structured assessment conducted by an unfamiliar adult to the individual being assessed. Separate Calibrated Severity Scores (CSS) based on the overall (ADOS CSS), social affect (SA CSS) and restricted and repetitive behaviour (RRB CSS) domains were also derived for each module [17, 35, 36]. ADOS-2 assessments were conducted by research members who had undertaken ADOS-2 for research training and had demonstrated > 80% coding reliability across all five modules.

### Procedure

Participants attended an appointment for psychological assessment and venous blood collection. All procedures were approved by The Royal Children’s Hospital and INTA Human Research Ethics Committees (HREC #33066 and #15, respectively). All parents/caregivers provided written informed consent and those who were deemed cognitively able also provided written informed consent.

### Data analysis

Summary statistics were presented by sample size and percentage for categorical variables and mean and standard deviation for continuous variables. Comparisons for the differences between sex for mean age and proportion of full mutation alleles only (FM-only) were carried out using a two-sample *t* test. Spearman’s rank correlation was used to assess relationships between *FMR1* mRNA and age in males and females.

For intellectual functioning and autism features, regression methods were used to compare the difference between sex or allelic class, or relationship with *FMR1* mRNA, adjusted for the covariates of age, country, ADOS CSS for intellectual functioning and corrected full scale IQ (cFSIQ) for autism features where significant. The semi-parametric regression was employed with age as the non-parametric component and other covariates as parametric components because intellectual functioning scores had a non linear relationship with age. Otherwise, either least square or robust regression (to downweight the effect of outliers) were used if age was not associated with the outcome variable.

To further examine the impact of *FMR1* mRNA levels on phenotypic variables in the male cohort, the FM-only group was split based on the presence (*FMR1* mRNA a.u. > 0) or absence (*FMR1* mRNA a.u. = 0) of *FMR1* mRNA in peripheral blood and comparisons were made between these two groups using semi-parametric regression for intellectual functioning scores and robust regression for ADOS scores. These same analyses were undertaken using only children and adolescents (< 19 years) and pre-pubertal children (< 13 years).

False discovery rate (FDR) was used to adjust for multiple testing. All analyses were carried out using commercial software Stata *version 15* (http://www.stata.com); *p* values were two-sided and a variable was considered significant if it was less than 0.05.

## Results

Male and female participants did not differ significantly on age (males: Mean (*M*) = 13.38, Standard Deviation (*SD*) = 10.07; females: *M* = 10.87, *SD* = 8.71; *p* = 0.244). Allelic classification (FM-only versus PM/FM mosaic) also did not differ between males and females (*p* = 0.672).

### Inter-group comparisons of intellectual functioning and autism features

Comparisons of phenotypic variables between males and females (FM-only and PM/FM mosaics combined) showed that males had significantly lower intellectual functioning scores on all domains assessed (Table 1 and Additional file 1: Table S1), though the two groups did not differ on ADOS scores, after controlling for cFSIQ (Table 1).

**Table 1:** Comparison between males and females on intellectual functioning (corrected) scores and autism features

	Males		Females		*p*
	*n*	*M* ± *SD*	*n*	*M* ± *SD*	
Intellectual functioning^a^					
cVIQ	66	42.5 ± 23.6	29	73.1 ± 17.6	< *0*.0001*
cPIQ	67	42.7 ± 19.7	29	66.9 ± 15.8	< *0*.0001*
cFSIQ	66	29.6 ± 24.2	29	65.9 ± 17.2	< *0*.0001*
Autism features^b^					
ADOS CSS	62	6.69 ± 2.00	25	4.92 ± 2.36	0.573
SA CSS	62	6.56 ± 2.19	25	4.80 ± 2.25	0.814
RRB CSS	62	7.37 ± 1.88	25	6.76 ± 2.28	0.708

^a^Semi-parametric regression adjusted for country, age and ADOS CSS

^b^Robust regression adjusted for country and cFSIQ; *p value remained < 0.05 after adjustment for multiple testing

### Inter-group comparisons of *FMR1* mRNA levels

**Fig. 1:**
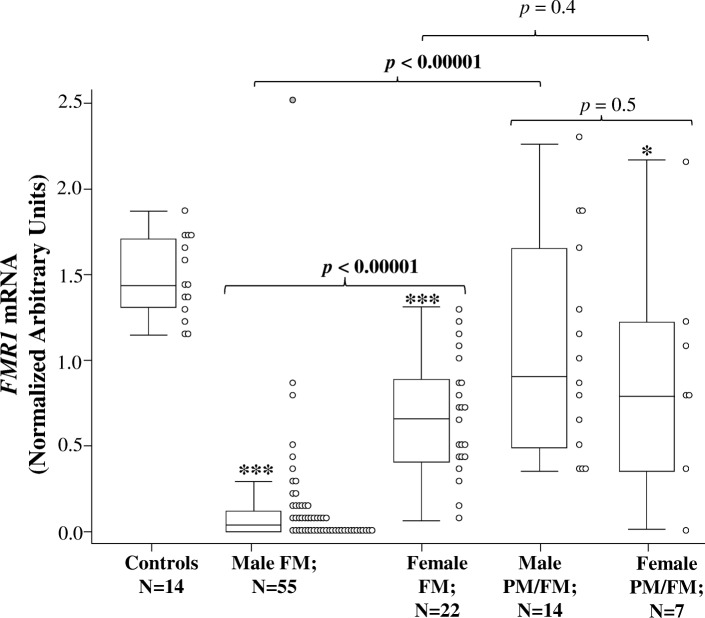
Intergroup comparisons of *FMR1* mRNA levels in blood of males and females with FXS and controls (CGG < 45). *p* values for the intergroup comparisons were derived using the Wilcoxon rank-sum test. *p < 0.05 compared to controls; ***p < 0.00001 compared to controls

FM-only males had significantly lower *FMR1* mRNA compared to FM-only females and PM/FM mosaic males (Fig. 1). The two FXS female groups did not significantly differ on *FMR1* mRNA levels and neither did the male and female PM/FM mosaic groups (Fig. 1). No females with FXS and no PM/FM mosaic males had completely silent *FMR1* mRNA (Females: 0.013–2.170 a.u.; PM/FM mosaic males: 0.354–2.260 a.u.), while 40% of FM-only males had complete silencing of *FMR1* mRNA (range 0.0–2.514 a.u.).

### Stratification based on presence or absence of complete *FMR1* mRNA silencing in FM-only males and associations with intellectual functioning and autistic features

**Fig. 2:**
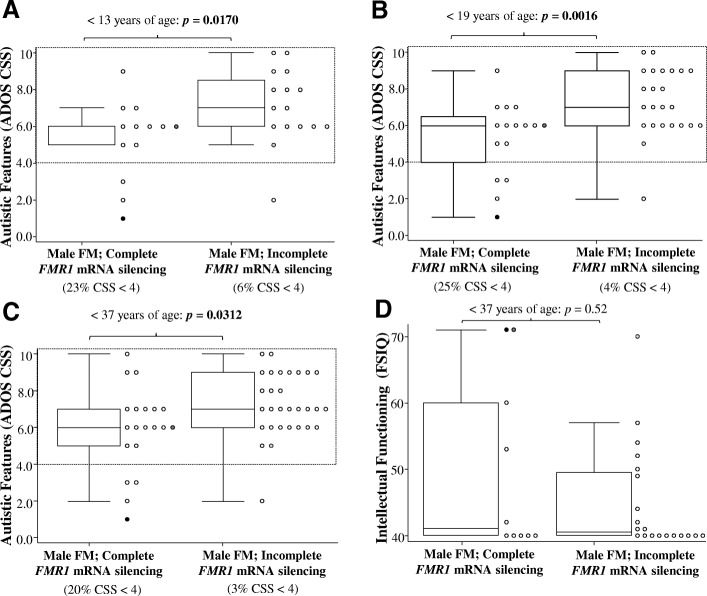
Autistic features and intellectual functioning in FM only males stratified based on presence or absence of *FMR1* mRNA in blood. Intergroup comparisons of autistic features in FM only males < 13 years of age (**a**), < 19 years of age (**b**), < 37 years of age (**c**) and intellectual functioning in FM only males < 37 years of age (**d**), with broken lines representing CSS scores ≥ 4 (ASD cut-off previously reported for ADOS-2 [37]). *p* values for the intergroup comparisons were derived using the Wilcoxon rank-sum test. Black, dark and light grey dots represent the same participants between all four figures with FSIQ ≥ 70

Comparisons between FM-only males with complete versus incomplete *FMR1* mRNA silencing demonstrated that FM-only males with incomplete *FMR1* mRNA silencing had significantly elevated ADOS CSS compared to FM-only males with complete *FMR1* mRNA silencing, for participants less than 19 years of age (Fig. 2b; *p* = 0.0016), and this difference remained significant after adjusting for country and cFSIQ and FDR, as did the difference on SA CSS (Table 2). When only children (< 13 years), or when adults were also included (< 37 years of age), ADOS CSS were also significantly higher in the incomplete silencing group (Fig. 2a,c). These differences were significant after adjusting for cFSIQ and country, but did not survive FDR (Table 2).

**Table 2:** Comparison between FM-only males with complete and incomplete *FMR1* mRNA silencing on intellectual functioning (corrected) scores and autism features

	FM-only with incomplete *FMR1* mRNA silencing			FM-only with complete *FMR1* mRNA silencing			*p*
	*n*	*M*	*SD*	*n*	*M*	*SD*	
Intellectual Functioning^a^							
cVIQ	33	37.0	26.9	21	41.5	20.4	0.196
cPIQ	33	36.8	20.6	21	44.4	19.5	0.980
cFSIQ	33	22.9	24.3	21	28.4	23.2	0.497
Autism features (whole sample)^b^							
ADOS CSS	30	7.23	1.74	20	5.90	2.29	*0*.*041*
SA CSS	30	7.07	2.00	20	5.95	2.56	*0*.*035*
RRB CSS	30	7.73	1.57	20	6.70	2.30	0.202
Autism features (< 19 years)^b^							
ADOS CSS	24	7.29	1.85	16	5.31	2.09	*0*.011*
SA CSS	24	7.08	2.19	16	5.50	2.61	*0*.029*
RRB CSS	24	7.83	1.66	16	6.50	2.42	0.171
Autism features (< 13 years)^b^							
ADOS CSS	19	7.16	1.92	13	5.31	2.18	*0*.*050*
SA CSS	19	7.11	2.21	13	5.15	2.58	*0*.*047*
RRB CSS	19	7.68	1.77	13	6.77	2.45	0.647

^a^Semi-parametric regression adjusted for country, ADOS CSS and age

^b^Robust regression adjusted for country and cFSIQ; *p value remained < 0.05 after adjustment for multiple testing

Italic values indicate significance prior to adjustment for multiple testing

Moreover, only 3% of FM-only males with incomplete *FMR1* mRNA silencing had an ADOS CSS that was less than 4, as compared to 20% of FM-only males with complete *FMR1* silencing (Fig. 2c); similar proportions of FM-only males with ADOS CSS below 4 were found when using children and adolescents (Fig. 2b) and children < 13 years (Fig. 2a). A CSS of 4 or above is the cut-off previously used for classification for presence of autism spectrum disorder (ASD) [37]. In contrast, the two FM-only male groups did not differ significantly on any of the intellectual functioning scores (Table 2 and Fig. 2d). No significant differences were also observed on intellectual functioning scores between the two FM-only groups when stratified by age groups (*p* > 0.05; Additional file 1: Table S2).

### Relationships between *FMR1* mRNA levels, age and phenotypes

**Fig. 3:**
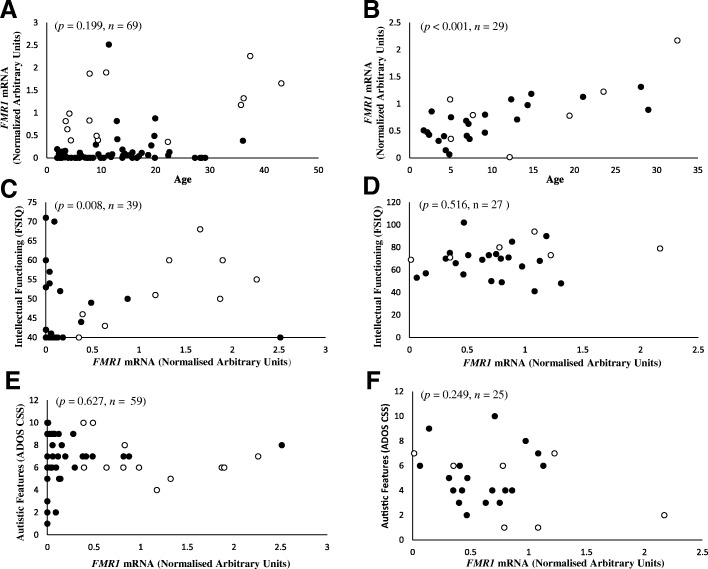
Relationships between age and phenotype severity with *FMR1* mRNA levels in blood of males and females with FXS. Relationships between *FMR1* mRNA levels in blood and age in (**a**) males and (**b**) females with FXS. Relationships between *FMR1* mRNA levels in blood and intellectual functioning in (**c**) FXS males using robust regression and (**d**) FXS females using ordinary regression (also see Table 3). Relationships between *FMR1* mRNA levels in blood and autistic features in (**e**) FXS males using robust regression adjusted for country and cFSIQ and (**f**) FXS females using robust regression adjusted for cFSIQ (also see Table 3). Solid dots represent FM-only, while open dots represent PM/FM mosaics

*FMR1* mRNA levels were positively correlated with age in FXS females (*n* = 29, correlation (*r*_*s*_) = 0.642, *p* < 0.001), but not in FXS males (*n* = 69, *r*_*s*_ = 0.157, *p* = 0.199; Fig. 3a, b). In males, regardless of allelic classification (FM-only and PM/FM mosaic), *FMR1* mRNA levels were significantly associated with corrected verbal IQ (cVIQ) and cFSIQ (Table 3). *FMR1* mRNA levels were significantly associated with all standard intellectual functioning scores in males (Additional file 1: Table S3; Fig. 3c). However, no associations were observed between *FMR1* mRNA and autistic features in the combined male FXS cohort (Table 3; Fig. 3e). When the WISC-III (Chile) and MSEL were removed, cVIQ and cFSIQ remained significantly associated with *FMR1* mRNA levels in males (Additional file 1: Table S4).

**Table 3:** Relationship between intellectual functioning (corrected) scores and autism features, with *FMR1* mRNA levels in males and females

	Males			Females		
	*n*	*β ± se*	*p*	*n*	*β ± se*	*p*
Intellectual Functioning (corrected)^a^						
cVIQ	66	9.09 *±* 3.90	*0*.023*	28	10.6 *±* 6.18	0.087
cPIQ	67	8.71 *±* 4.77	0.072	28	2.85 *±* 4.68	0.543
cFSIQ	66	9.46 *±* 4.24	*0*.029*	28	5.32 *±* 4.50	0.237
Autism features^b^						
ADOS CSS	59	0.12 *±* 0.25	0.627	25	− 8.00 *±* 0.69	0.249
SA CSS	59	0.52 *±* 0.33	0.118	25	− 0.78 *±* 0.71	0.270
RRB CSS	59	− 0.13 *±* 0.25	0.612	25	0.22 *±* 0.69	0.753

^a^Semi-parametric regression adjusted for country, ADOS CSS and age for males and robust regression adjusted for ADOS CSS for females

^b^Robust regression adjusted for country and corrected FSIQ for males and only corrected FSIQ for females; *β* = estimated regression coefficient; se = standard error. *p value remained < 0.05 after adjustment for multiple testing

Italic values indicate significance prior to adjustment for multiple testing

In contrast, *FMR1* mRNA levels were not associated with any of the corrected intellectual functioning scores or ADOS CSS in FXS females (Table 3; Fig. 3d, f). Similarly, no associations were observed between *FMR1* mRNA levels and standard intellectual functioning scores (Additional file 1: Table S3) or corrected intellectual functioning scores when the WISC-III (Chile) and MSEL were removed (Additional file 1: Table S4) in females with FXS.

### Associations between *FMR1* mRNA levels and intellectual functioning and autistic features in FM-only males with incomplete *FMR1* mRNA silencing and PM/FM mosaic males

Significant associations were observed with cVIQ, corrected performance IQ (cPIQ) and cFSIQ (Table 4) with *FMR1* mRNA levels in the PM/FM mosaic group. VIQ, PIQ and FSIQ were associated with *FMR1* mRNA levels in both FM-only males with incomplete silencing and PM/FM mosaic males (Additional file 1: Table S5). The association between *FMR1* mRNA levels and standard FSIQ in the combined cohort was predominantly due to the associations being observed in the PM/FM mosaic males, and not the FM-only male group (Fig. 3c). In contrast, no significant associations were found between *FMR1* mRNA levels and autistic features in these two groups of males (Table 4).

**Table 4:** Relationship between intellectual functioning (corrected) scores, and autism features, with *FMR1* mRNA levels in FM-only males with incomplete *FMR1* mRNA silencing and PM/FM mosaic males

	FM-only with incomplete *FMR1* mRNA silencing			PM/FM mosaic		
	*n*	*β ± se*	*p*	*n*	*β ± se*	*p*
Intellectual functioning						
cVIQ	32	14.7 *±* 38.7	0.705	12	13.6 *±* 4.52	*0*.003*
cPIQ	32	13.5 *±* 25.3	0.594	13	14.8 *±* 3.77	< *0*.001*
cFSIQ	32	6.75 *±* 27.6	0.807	12	9.62 *±* 4.28	*0*.025*
Autism features						
ADOS CSS	29	− 0.78 *±* 0.77	0.310	12	− 0.85 *±* 0.87	0.331
SA CSS	29	− 0.60 *±* 0.98	0.538	12	− 0.79 *±* 1.04	0.448
RRB CSS	29	− 0.92 *±* 0.96	0.338	12	− 1.16 *±* 0.65	0.074

Robust regression was used to conduct analysis, without adjustment for any covariate. *p value remained < 0.05 after adjustment for multiple testing

Italic values indicate significance prior to adjustment for multiple testing

## Discussion

This is the first study to demonstrate that the presence of incomplete *FMR1* mRNA silencing in blood is significantly associated with more severe autism features (predominantly social communication difficulties) in FM-only males. Specifically, FM-only males with incomplete *FMR1* mRNA silencing had elevated ADOS CSS and SA CSS compared to those with complete *FMR1* mRNA silencing, though these associations were predominantly observed in the paediatric cohort (< 19 years). Moreover, 21% more of the FM-only male group with incomplete silencing aged < 19 years met the ADOS-2 criteria for ASD (CSS ≥ 4), as compared to FM-only males with complete *FMR1* mRNA silencing (< 19 years). In contrast, there were no differences on intellectual functioning scores (e.g. FSIQ), when stratified based on the presence of FM *FMR1* mRNA in blood. These findings suggest that the link between presence of potentially toxic FM mRNA and autism features may be stronger during earlier development and adolescence before neurodegenerative processes begin in adulthood that may confound the analysis. The loss of significant differences post FDR in the male children aged under 13 years may be an artefact of the smaller sample size or indeed that adolescence is a critical time-point.

Intellectual disability and autistic features, while linked, are variable in FXS, which may be explained by contribution from two different, but overlapping, patho-mechanisms: (i) namely silencing of mRNA resulting in loss of FMRP; and (ii) overexpression of toxic expanded mRNA by active unmethylated FM alleles. This is particularly important in light of the significant research performed to date aiming to re-activate methylated FM alleles as a potential therapy performed in various cell line models [38–42]. This may also explain, in part, why preclinical studies in *FMR1* knockout (KO) animal models, trialling drugs that correct pathways dysregulated due to complete loss of *FMR1*, were largely unsuccessful in human trials [43]. The *FMR1* FM mRNA toxicity mechanism does not apply to *FMR1* KO animal models, while it may have significant implications for the behavioural phenotype in patients affected with FXS, especially because behavioural phenotypes were targeted by most primary outcome measures in the recent clinical trials [43, 44]. If FM mRNA is indeed expressed in the majority of FXS males, consistent with the results for 60% of FM-only males in this study, even at low levels, reactivation of large expanded alleles may not be a viable in vivo treatment strategy. While increase in FM mRNA and presence of FMRP may lead to improvements in intellectual functioning in males with unmethylated FM alleles, issues associated with RNA toxicity may be exacerbated. Perhaps excision of FM all together, as previously described [45, 46], or treatment of downstream pathways targeting both FM RNA toxicity as well as FMRP deficiency is a better option.

Since FXTAS is believed to be associated with RNA toxicity, and previous adult patients with FXS and FXTAS features had low bulk (average levels between cells) *FMR1* mRNA levels [11–13] similar to those observed in this study, our data suggests that expression of expanded FM alleles may be toxic, independent of high or low bulk mRNA levels, as compared to the control range. The stratification based on the presence or absence of FM mRNA reported in this study also suggests that potentially toxic mRNA expressed from FM alleles may be implicated in the pathogenesis of autism, though further studies in larger FXS cohorts and functional studies are required to confirm these findings.

It is notable that the RNA toxicity hypotheses cannot be explored in the current KO mouse models of FXS and animal models do not fully recapitulate the behavioural phenotype of FXS. An alternative approach to explore in vivo functional links between mRNA toxicity, the type of toxicity (e.g. including toxic gain of function, as well as sense and anti-sense Repeat Associated non-ATG translation [Reviewed in Kraan et al. [2]]) and FMRP deficiency would be to use post-mortem brain tissues from FXS males, who had previously undergone formal assessments of intellectual functioning and autism.

Three cases in the present study (highlighted in Fig. 2) further demonstrate the complexity of disentangling the relationships between autism and ID in FXS. Two cases in the FM-only group with complete *FMR1* mRNA silencing had FSIQ scores of 71 and while one of these cases had moderate autism features (ADOS CSS = 6), the other had minimal autism features (ADOS CSS = 1); these cases are represented by the dark grey and black dots in Fig. 2, respectively. The third case, from the incomplete silencing group, had a FSIQ of 70 and minimal autism features (ADOS CSS = 2). This latter participant had an *FMR1* mRNA level of 0.091 a.u. and is represented by the light grey dot in Fig. 2. This low level of *FMR1* expression in these three higher functioning FXS cases suggests that blood may have limited representation of *FMR1* mRNA levels in the brain in a proportion of cases; though other factors may also contribute. Increasing age has been associated with increasing autism symptoms in males with FXS [47, 48], while a widening gap between FXS and typically developing individuals has been observed for intellectual functioning measures [31]. Of note is that these three cases were aged 3.92, 5.49 and 2.21 years, respectively.

Despite the significant differences observed between the two complete and incomplete *FMR1* mRNA FM-only groups on ADOS CSS, the overlap between autism features and anxiety, particularly social anxiety, cannot be ignored. It has been suggested that elevated symptoms of ASD in FXS can be attributed to the social anxiety experienced by these individuals [49, 50]. Roberts and colleagues [49] demonstrated significant associations between a composite social anxiety measure (derived from the Social Avoidance Scale [51, 52], Anxiety Depression and Mood Scale: Avoidance subscale [53] and the Child Behavior Checklist-Anxiety problems scale [54]) and ADOS CSS in a sample of 59 adolescent and adult FXS males (15–23 years), highlighting the significant overlap between ASD features and social anxiety in FXS. Thus, it is possible that expression of *FMR1* mRNA is associated with social anxiety rather than ASD features, or a combination of overlapping symptoms between the two constructs. As concluded by Roberts and colleagues [49], the relationship between social anxiety and ADOS-2 scores is likely bi-directional. Future research aiming to disentangle autism features and social anxiety and their relationship with molecular mechanisms, such as *FMR1* mRNA, is warranted, as this may have implications for treatments that target these specific behaviours.

If confirmed in future studies that FM mRNA is associated with autism features, then stratification of FM males based on the presence or absence of mRNA may be a complimentary approach to define the presence of mosaicism in FXS males, especially for clinical trials where primary outcome measures target behavioural issues. Such patient stratification has previously been shown to result in different treatment outcomes [55]. In the placebo controlled trial of AFQ056 (mavoglurant), a mGluR5 antagonist targeting FMRP deficiency, in 30 males with FXS, no significant effects of treatment were observed on Aberrant Behavior Checklist-Community (ABC-C) [56] total scores when males with complete and incomplete *FMR1* mRNA silencing were analysed as one combined group. However, significant improvements in ABC-C scores were observed in all patients who had complete absence of *FMR1* mRNA (*n* = 7), after treatment compared to placebo [55].

Only one study has specifically explored the relationship between *FMR1* mRNA levels in blood and autism features in males with FXS (*n* = 63; 38% PM/FM mosaic), and provided appropriate statistical analyses at group level [3]. This study demonstrated that none of the molecular markers analysed, including *FMR1* mRNA in blood, were associated with communication-social total scores of the ADOS or with the overall autism rating. Interestingly, the male with the most elevated mRNA level in that cohort had autism, prompting the researchers to suggest further research in this area [3]. The study, however, did not dichotomize FXS participants based on the presence of complete or incomplete *FMR1* silencing, and did not report on whether this stratification strategy resulted in associations with elevated autism features in FXS, as reported in this study.

Moreover, the Harris et al. [3], and most of the studies that followed examined *FMR1* mRNA “toxicity”, using the real-time PCR method targeting a single 5′ region of *FMR1* mRNA [57]. The levels detected by this single assay were then normalised to β-glucuronidase as a single internal control gene in most of these studies, described in Kraan et al. [14]. Importantly, Kraan et al. [14] reported that β-glucuronidase expression was not stably expressed in blood, and has been itself associated with PM-related phenotypes [14, 15]. In contrast, this study used a real-time PCR method that targeted two conserved regions at both 5′ and 3′ends of *FMR1* mRNA, normalising the target mRNA levels to multiple internal control genes [15, 16]. This was an improved methodology that allowed for a more accurate quantification of mRNA because targeting mRNA at both ends by this approach controls for technical variability resulting from loss of signal due to mRNA degradation at either end of the mRNA [58]. This approach at the same time normalised the mRNA levels detected by the target assays to multiple stably expressed control genes, that have not been previously associated with PM or any other *FMR1* related phenotypes [15, 16].

Using this highly quantitative method in the current study, significant associations were also observed between *FMR1* mRNA levels in blood and intellectual functioning scores in FXS males. The most consistent findings were poorer verbal abilities and overall intellectual functioning associated with lower *FMR1* mRNA. Nonetheless, these associations were primarily driven by the PM/FM mosaic cohort. When examining the associations between *FMR1* mRNA and corrected intellectual functioning in the FM-only male group with incomplete *FMR1* mRNA silencing, no significant associations were found, while associations were consistently observed in the PM/FM mosaic male group.

As expected, females with FXS had significantly higher levels of *FMR1* mRNA and less severe phenotype, than males. However, in females, there were no significant associations between *FMR1* mRNA and intellectual functioning and autistic features. In contrast, there was a highly significant association between mRNA levels and age, in females but not males, whereby older age was associated with higher *FMR1* mRNA levels. This is consistent with previous literature examining *FMR1* promoter methylation in FXS, where methylation decreased with age in females (but not males) [59]. This supports the previous hypothesis that in blood of FM females, there is likely positive selection for cells with the normal CGG size allele on the active X over time, thus leading to increased *FMR1* expression with age.

Interestingly, this increase in mRNA with age does not appear to be associated with improvements in the cognitive and behavioural phenotypes in the females included in this study. This lack of association in females, but not males, may be explained by a significantly smaller sample size of FXS females as compared to males, in this study or potentially because expression in blood at a single time-point (where there is a high turnover of cells) may not reflect expression in the brain, where cell turnover is not as high. Moreover, the cross-sectional analysis of this data does not allow for observation of change over time, including the interplay of biological drivers (e.g. *FMR1* mRNA), environmental factors and the phenotype. Thus, longitudinal studies are required to further explore these findings.

Considering that FM *FMR1* mRNA is expressed in most FXS males, future studies should also explore expression of antisense transcripts including *ASFMR1* and *FMR4* previously reported to be over-expressed in PM-related disorders [21]. Analysis of expanded CGG repeat associated sense and antisense non-AUG translation, previously associated with PM-related disorders as an alternative mechanism to RNA toxicity, and relationships between these variables and FXS phenotypes, would also be of great interest in future FXS studies.

### Limitations

Notwithstanding its strengths, an important limitation of this study is that the study reports *FMR1* mRNA levels assuming that they correspond to protein (FMRP) levels, with the latter being the ultimate “top level” molecular factor in FXS. While this is a solid assumption for FM-only males with reported complete silencing, where FMRP could not be present with no mRNA to translate, for FM males with incomplete silencing, levels of *FMR1* mRNA may not reflect those of FMRP. Future studies should explore if stratification of FXS males based on the presence or absence of FMRP is concordant with the findings reported in this study using *FMR1* mRNA-based stratification.

Another limitation is that *FMR1* mRNA analysis performed using real-time PCR represents bulk mRNA levels. As previously suggested in studies examining *FMR1* mRNA toxicity in mosaic FXS cases with FXTAS phenotypes [60, 61], bulk mRNA analysis averages out levels between cells that overexpress mRNA and cells that have mRNA completely silenced. This may mean that if there is a small number of cells expressing toxic/elevated FM *FMR1* mRNA, and the majority have mRNA completely silenced, the bulk mRNA result will be decreased total mRNA output. This may also explain the lack of associations observed between *FMR1* mRNA levels and autism features in the FM-only male group with incomplete silencing, while group differences on ADOS CSS when stratified based on the presence or absence of *FMR1* mRNA were present. Future studies should explore the utility of single-cell RNA sequencing to further detangle the *FMR1* silencing and FM mRNA toxicity mechanisms in FXS at a single cell level.

Another limitation of the current study is the small sample sizes for the PM/FM mosaic male and female cohorts, which limits the generalisability of the findings in these groups. The use of multiple assessment types for intellectual functioning is common in FXS; however, the MSEL is qualitatively different to the Wechsler scales and the WISC-III (Chilean edition) incorporates PS and WM tasks in the calculation of VIQ and PIQ, which may impact scores. Nonetheless, when those individuals who were assessed with these measures were removed from the analyses, similar results were observed. The use of the Stanford Binet Intelligence Scales-Fifth edition [62] may be a more suitable measure in future studies where wide age ranges can be used. Lastly, only female control data is included in this study for reference ranges of *FMR1* mRNA; however, these levels overlap with age-matched males that have previously been reported elsewhere [63].

Future studies should aim to expand upon and replicate the findings of the current study by (i) recruitment of larger independent cohorts, including increasing the sample size within each age bracket; (ii) undertaking longitudinal studies that examine developmental trajectories, particularly in the transition from childhood to adolescence and then again in the transition from adolescence to adulthood; (iii) incorporate a comprehensive multi-disciplinary assessment of ASD based on DSM-5 criteria to determine whether similar findings are observed for those with a comorbid clinical diagnosis of ASD; (iv) including a measure of social anxiety to tease apart autism features and anxiety; and (v) include other molecular markers such as FMRP. Such research will aid in understanding how molecular mechanisms (e.g. *FMR1* mRNA and FMRP) relate to specific clinical features of FXS across the lifespan.

## Conclusion

In summary, this study characterised a large international FXS cohort, demonstrating that presence of *FMR1* transcription in FM-only males is common (present in 60% of FM-only males), and is associated with more severe autism features, including social communication difficulties, but not intellectual functioning (reflected by FSIQ). On the one hand, incomplete silencing of *FMR1* was associated with elevated autistic features as measured by ADOS-2 CSS, with 21% more of the 18 and under FM-only group expressing FM *FMR1* mRNA meeting the ADOS-2 criteria for ASD (CSS ≥ 4), as compared to FM-only males with completely silenced *FMR1*. On the other hand, decreased levels of *FMR1* mRNA were associated with decreased intellectual functioning in FXS males, with the relationships primarily driven by variability in the PM/FM mosaic subgroup. These novel findings, if confirmed by future independent studies, indicate that silencing of mRNA resulting in loss of FMRP, and overexpression of toxic expanded *FMR1* FM mRNA, occur together in the same individuals, for most FXS males. We postulate that the two reciprocal mechanisms may contribute to different, but overlapping aspects of FXS, namely intellectual disability and autism phenotype. This may have implications for (i) patient stratification in clinical trials and the outcome measures used in stratified subgroups; (ii) design of treatment strategies aiming to re-activate *FMR1* in FXS, which may result in production of harmful FM mRNA by a small proportion of cells; and (iii) preclinical trials targeting downstream pathways to both FM RNA toxicity as well as FMRP deficiency, as opposed to widely used, FXS KO models, where FM RNA toxicity cannot be studied.

## Additional file

Additional file 1: **Table S1.** Comparison between males and females intellectual functioning (standard) scores. **Table S2.** Comparison between FM-only males with complete and incomplete *FMR1* mRNA silencing on intellectual functioning (corrected) scores and autism features. **Table S3.** Relationship between intellectual functioning (standard) scores and *FMR1* mRNA in males and females. **Table S4.** Relationship between intellectual functioning scores (corrected) and *FMR1* mRNA in males and females with WISC-III (Chile) and MSEL removed. **Table S5.** Relationship between intellectual functioning (standard) scores with *FMR1* mRNA in FM-only males with incomplete *FMR1* mRNA silencing and PM/FM mosaic males. (DOCX 27 kb)

## Abbreviations

ADOS CSS: Overall ADOS-2 Calibrated Severity Score
ADOS: Autism Diagnostic Observation Schedule
ADOS-2: Autism Diagnostic Observation Schedule-2nd Edition
ASD: Autism spectrum disorder
cFSIQ: Corrected full scale IQ
cPIQ: Corrected performance IQ
CSS: Calibrated severity score
cVIQ: Corrected verbal IQ
FDR: False discovery rate
FM: Full mutation
FM-only: Full mutation alleles only
FMRP: Fragile X mental retardation protein
FSIQ: Full Scale IQ
FXS: Fragile X syndrome
KO: Knockout
MSEL: Mullen Scales of Early Learning
PIQ: Performance IQ
PM: Premutation
PM/FM: Premutation/Full mutation mosaic
RRB CSS: Repetitive and Restricted Behaviour Calibrated Severity Scores
SA CSS: Social Affect Calibrated Severity Scores
VIQ: Verbal IQ
WAIS-IV: Wechsler Adult Intelligence Scale-Fourth Edition
WISC-III: Wechsler Intelligence Scale for Children-Third Edition
WISC-IV: Wechsler Intelligence Scale for Children-Fourth Edition
WPPSI-III: Wechsler Preschool and Primary Scale of Intelligence-Third Edition

## References

[1] Verkerk AJ, Pieretti M, Sutcliffe JS, Fu YH, Kuhl DP, Pizzuti A (1991). Identification of a gene (FMR-1) containing a CGG repeat coincident with a breakpoint cluster region exhibiting length variation in fragile X syndrome. Cell..

[2] Kraan CM, Godler DE, Amor DJ (2019). Epigenetics of fragile X syndrome and fragile X-related disorders. Dev Med Child Neurol.

[3] Harris SW, Hessl D, Goodlin-Jones B, Ferranti J, Bacalman S, Barbato I (2008). Autism profiles of males with fragile X syndrome. Am J Ment Retard.

[4] Rodriguez-Revenga L, Madrigal I, Badenas C, Xuncla M, Jimenez L, Mila M (2009). Premature ovarian failure and fragile X female premutation carriers: no evidence for a skewed X-chromosome inactivation pattern. Menopause..

[5] Sherman SL (2000). Premature ovarian failure in the fragile X syndrome. Am J Med Genet.

[6] Rousseau F, Heitz D, Biancalana V, Blumenfeld S, Kretz C, Boue J (1991). Direct diagnosis by DNA analysis of the fragile X syndrome of mental retardation. N Engl J Med.

[7] Nolin SL, Glicksman A, Houck GE, Brown WT, Dobkin CS (1994). Mosaicism in fragile X affected males. Am J Med Genet.

[8] Aliaga SM, Slater HR, Francis D, Du Sart D, Li X, Amor DJ (2016). Identification of males with cryptic fragile X alleles by methylation-specific quantitative melt analysis. Clin Chem.

[9] Rousseau F, Heitz D, Tarleton J, MacPherson J, Malmgren H, Dahl N (1994). A multicenter study on genotype-phenotype correlations in the fragile X syndrome, using direct diagnosis with probe StB12.3: the first 2,253 cases. Am J Hum Genet.

[10] Hernandez RN, Feinberg RL, Vaurio R, Passanante NM, Thompson RE, Kaufmann WE (2009). Autism spectrum disorder in fragile X syndrome: a longitudinal evaluation. Am J Med Genet A.

[11] Santa María L, Pugin A, Alliende MA, Aliaga S, Curotto B, Aravena T (2014). FXTAS in an unmethylated mosaic male with fragile X syndrome from Chile. Clin Genet.

[12] Pretto DI, Hunsaker MR, Cunningham CL, Greco CM, Hagerman RJ, Noctor SC (2013). Intranuclear inclusions in a fragile X mosaic male. Transl Neurodegener.

[13] Loesch DZ, Sherwell S, Kinsella G, Tassone F, Taylor A, Amor D (2012). Fragile X-associated tremor/ataxia phenotype in a male carrier of unmethylated full mutation in the FMR1 gene. Clin Genet.

[14] Kraan CM, Cornish KM, Bui QM, Li X, Slater HR, Godler DE (2016). β-glucuronidase mRNA levels are correlated with gait and working memory in premutation females: understanding the role of FMR1 premutation alleles. Sci Rep.

[15] Kraan CM, Cornish KM, Bui QM, Li X, Slater HR, Godler DE (2018). β-glucuronidase use as a single internal control gene may confound analysis in FMR1 mRNA toxicity studies. PloS one.

[16] Shelton AL, Cornish KM, Kolbe S, Clough M, Slater HR, Li X (2016). Brain structure and intragenic DNA methylation are correlated, and predict executive dysfunction in fragile X premutation females. Transl Psychiatry.

[17] Hus V, Gotham K, Lord C (2014). Standardizing ADOS domain scores: separating severity of social affect and restricted and repetitive behaviors. J Autism Dev Disord.

[18] Cornish KM, Kraan CM, Bui QM, Bellgrove MA, Metcalfe SA, Trollor JN (2015). Novel methylation markers of the dysexecutive-psychiatric phenotype in FMR1 premutation women. Neurology..

[19] Francis D, Burgess T, Mitchell J, Slater H (2000). Identification of small FRAXA premutations. Mol Diagn.

[20] Alliende MA, Urzua B, Valiente A, Cortes F, Curotto B, Rojas C (1998). Direct molecular analysis of FMR-1 gene mutation in patients with fragile Xq syndrome and their families. Rev Med Chil.

[21] Loesch DZ, Godler DE, Evans A, Bui QM, Gehling F, Kotschet KE (2011). Evidence for the toxicity of bidirectional transcripts and mitochondrial dysfunction in blood associated with small CGG expansions in the FMR1 gene in patients with parkinsonism. Genet Med.

[22] Mullen EM. Mullen scales of early learning. Circle Pines: American Guidance Service; 1995.

[23] Wechsler D. Wechsler preschool and primary scale of intelligence-third edition Australian standardised edition. Sydney: NCS Pearson Inc.; 2004.

[24] Wechsler D. Wechsler preschool and primary scale of intelligence-third edition Mexican edition. Mexico: Harcourt Assessment; 2002.

[25] Wechsler D. Wechsler intelligence scale for children–fourth edition Australian standardised edition. Sydney: NCS Pearson Inc.; 2003.

[26] Wechsler D. Wechsler intelligence scale for children-third edition Chilean edition. San Antonio: The Psychological Corporation; 2007.

[27] Wechsler D. Wechsler adult intelligence scale—fourth edition Australian and New Zealand language adaptation. Sydney: NCS Pearson Inc.; 2008.

[28] Wechsler D. Wechsler adult intelligence scale-fourth edition Chilean edition. San Antonio: NCS Pearson Inc; 2008.

[29] Arpone M, Baker EK, Bretherton L, Bui M, Li X, Whitaker S (2018). Intragenic DNA methylation in buccal epithelial cells and intellectual functioning in a paediatric cohort of males with fragile X. Sci Rep.

[30] Baker S, Hooper S, Skinner M, Hatton D, Schaaf J, Ornstein P (2011). Working memory subsystems and task complexity in young boys with fragile X syndrome. J Intellect Disabil Res.

[31] Quintin EM, Jo B, Hall SS, Bruno JL, Chromik LC, Raman MM (2016). The cognitive developmental profile associated with fragile X syndrome: a longitudinal investigation of cognitive strengths and weaknesses through childhood and adolescence. Dev Psychopathol.

[32] Bishop SL, Richler J, Lord C (2006). Association between restricted and repetitive behaviors and nonverbal IQ in children with autism Spectrum disorders. Child Neuropsychol.

[33] Richler J, Bishop SL, Kleinke JR, Lord C (2007). Restricted and repetitive behaviors in young children with autism spectrum disorders. J Autism Dev Disord.

[34] Lord C, Rutter M, DiLavore PC, Risi S, Gotham K, Bishop SL (2012). Autism diagnostic observation schedule, 2nd edition (ADOS-2).

[35] Hus V, Lord C (2014). The autism diagnostic observation schedule, module 4: revised algorithm and standardized severity scores. J Autism Dev Disord.

[36] Esler AN, Bal VH, Guthrie W, Wetherby A, Ellis Weismer S, Lord C (2015). The autism diagnostic observation schedule, toddler module: standardized severity scores. J Autism Dev Disord.

[37] Gotham K, Pickles A, Lord C (2009). Standardizing ADOS scores for a measure of severity in autism spectrum disorders. J Autism Dev Disord.

[38] Pietrobono R, Pomponi MG, Tabolacci E, Oostra B, Chiurazzi P, Neri G (2002). Quantitative analysis of DNA demethylation and transcriptional reactivation of the FMR1 gene in fragile X cells treated with 5-azadeoxycytidine. Nucleic Acids Res.

[39] Chiurazzi P, Pomponi MG, Willemsen R, Oostra BA, Neri G (1998). In vitro reactivation of the FMR1 gene involved in fragile X syndrome. Hum Mol Genet.

[40] Coffee B, Zhang F, Warren ST, Reines D (1999). Acetylated histones are associated with FMR1 in normal but not fragile X-syndrome cells. Nat Genet.

[41] Tabolacci E, Pietrobono R, Moscato U, Oostra BA, Chiurazzi P, Neri G (2005). Differential epigenetic modifications in the FMR1 gene of the fragile X syndrome after reactivating pharmacological treatments. Eur J Hum Genet.

[42] Bar-Nur O, Caspi I, Benvenisty N (2012). Molecular analysis of FMR1 reactivation in fragile-X induced pluripotent stem cells and their neuronal derivatives. J Mol Cell Biol.

[43] Berry-Kravis E, Des Portes V, Hagerman R, Jacquemont S, Charles P, Visootsak J (2016). Mavoglurant in fragile X syndrome: results of two randomized, double-blind, placebo-controlled trials. Sci Transl Med.

[44] Bailey DB, Berry-Kravis E, Wheeler A, Raspa M, Merrien F, Ricart J (2016). Mavoglurant in adolescents with fragile X syndrome: analysis of clinical global impression-improvement source data from a double-blind therapeutic study followed by an open-label, long-term extension study. J Neurodev Disord.

[45] Park CY, Halevy T, Lee DR, Sung JJ, Lee JS, Yanuka O (2015). Reversion of FMR1 methylation and silencing by editing the triplet repeats in fragile X iPSC-derived neurons. Cell Rep.

[46] Xie N, Gong H, Suhl JA, Chopra P, Wang T, Warren ST (2016). Reactivation of FMR1 by CRISPR/Cas9-mediated deletion of the expanded CGG-repeat of the fragile X chromosome. PLoS One.

[47] Thurman AJ, McDuffie A, Kover ST, Hagerman RJ, Abbeduto L (2015). Autism symptomatology in boys with fragile X syndrome: a cross sectional developmental trajectories comparison with nonsyndromic autism Spectrum disorder. J Autism Dev Disord.

[48] Lee M, Martin GE, Berry-Kravis E, Losh M (2016). A developmental, longitudinal investigation of autism phenotypic profiles in fragile X syndrome. J Neurodev Disord.

[49] Roberts JE, Ezell JE, Fairchild AJ, Klusek J, Thurman AJ, McDuffie A (2018). Biobehavioral composite of social aspects of anxiety in young adults with fragile X syndrome contrasted to autism spectrum disorder. Am J Med Genet B Neuropsychiatr Genet.

[50] Cordeiro L, Ballinger E, Hagerman R, Hessl D (2011). Clinical assessment of DSM-IV anxiety disorders in fragile X syndrome: prevalence and characterization. J Neurodev Disord.

[51] Roberts JE, Clarke MA, Alcorn K, Carter JC, Long AC, Kaufmann WE (2009). Autistic behavior in boys with fragile X syndrome: social approach and HPA-axis dysfunction. J Neurodev Disord.

[52] Roberts JE, Weisenfeld LA, Hatton DD, Heath M, Kaufmann WE (2007). Social approach and autistic behavior in children with fragile X syndrome. J Autism Dev Disord.

[53] Esbensen AJ, Rojahn J, Aman MG, Ruedrich S (2003). Reliability and validity of an assessment instrument for anxiety, depression, and mood among individuals with mental retardation. J Autism Dev Disord.

[54] Achenbach TM, Rescorla LA (2001). Manual for the ASEBA school-age forms & profiles.

[55] Jacquemont S, Curie A, des Portes V, Torrioli MG, Berry-Kravis E, Hagerman RJ (2011). Epigenetic modification of the FMR1 gene in fragile X syndrome is associated with differential response to the mGluR5 antagonist AFQ056. Sci Transl Med.

[56] Aman MG, Singh NN, Stewart AW, Field CJ (1985). The aberrant behavior checklist: a behavior rating scale for the assessment of treatment effects. Am J Ment Defic.

[57] Tassone F, Hagerman RJ, Chamberlain WD, Hagerman PJ (2000). Transcription of the FMR1 gene in individuals with fragile X syndrome. Am J Med Genet.

[58] Godler DE, Loesch DZ, Huggins R, Gordon L, Slater HR, Gehling F (2009). Improved methodology for assessment of mRNA levels in blood of patients with FMR1 related disorders. BMC Clin Pathol.

[59] Godler DE, Inaba Y, Shi EZ, Skinner C, Bui QM, Francis D (2013). Relationships between age and epi-genotype of the FMR1 exon 1/intron 1 boundary are consistent with non-random X-chromosome inactivation in FM individuals, with the selection for the unmethylated state being most significant between birth and puberty. Hum Mol Genet.

[60] Hwang YT, Dudding T, Aliaga SM, Arpone M, Francis D, Li X, et al. Molecular inconsistencies in a fragile X male with early onset ataxia. Genes. 2016;7(9):68. 10.3390/genes7090068 PMC5042398 27657133

[61] Hwang YT, Aliaga SM, Arpone M, Francis D, Li X, Chong B (2016). Partially methylated alleles, microdeletion, and tissue mosaicism in a fragile X male with tremor and ataxia at 30 years of age: a case report. Am J Med Genet.

[62] Gale HR (2003). Stanford Binet intelligence scales.

[63] Cvejic RC, Hocking DR, Wen W, Georgiou-Karistianis N, Cornish KM, Godler DE, et al. Reduced caudate volume and cognitive slowing in men at risk of fragile X-associated tremor ataxia syndrome. Brain Imaging Behav. 2018. 10.1007/s11682-018-9928-7 30046972

