# Supplementary material for: Incomplete silencing of full mutation alleles in males with fragile X syndrome is associated with autistic features
Source: Mol Autism. 2019 May 3;10:21. doi: 10.1186/s13229-019-0271-7 (PMC6499941; doi:10.1186/s13229-019-0271-7)
Supplement: Supplementary file 1 — Table S1. Comparison between males and females intellectual functioning (standard) scores. Table S2. Comparison between FM-only males with complete and incomplete FMR1 mRNA silencing on intellectual functioning (corrected) scores and autism features. Table S3. Relationship between intellectual functioning (standard) scores and FMR1 mRNA in males and females. Table S4. Relationship between intellectual functioning scores (corrected) and FMR1 mRNA in males and females with WISC-III (Chile) and MSEL removed. Table S5. Relationship between intellectual functioning (standard) scores with FMR1 mRNA in FM-only males with incomplete FMR1 mRNA silencing and PM/FM mosaic males. (DOCX 27 kb) [file 13229_2019_271_MOESM1_ESM.docx]

**Table S1:** Comparison between males and females intellectual functioning (standard) scores.

|  | **Males** | | **Females** | |  |
| --- | --- | --- | --- | --- | --- |
|  | *n* | *Mean* ± *SD*  (Range) | *n* | *Mean* ± *SD*  (Range) | *p* |
| **VIQ** | 47 | 57.3 ± 11.6  43 – 85 | 27 | 76.1 ± 15.4  47 – 110 | **<0.0001** |
| **PIQ** | 51 | 53.9 ± 10.6  31 – 81 | 28 | 69.3 ± 12.5  49 – 100 | **<0.0001** |
| **FSIQ** | 39 | 47.9 ± 10.0  40 – 71 | 27 | 69.2 ± 14.3  41 – 102 | **<0.0001** |

Robust regression was used to compare the difference between sex, adjusted for ADOS CSS. All p-values < 0.05 after adjustment for multiple testing.

**Table S2:** Comparison between FM males with complete and incomplete FMR1 mRNA silencing on intellectual functioning (corrected) scores and autism features.

|  | **FM-Only with incomplete *FMR1* mRNA silencing** | | | **FM-Only with complete *FMR1* mRNA silencing** | | |  |
| --- | --- | --- | --- | --- | --- | --- | --- |
|  | ***n*** | ***M*** | ***SD*** | ***n*** | ***M*** | ***SD*** | ***p*** |
| **Intellectual Functioning (≤ 18 years)** | | | | | | | |
| cVIQ | 26 | 30.65 | 26.25 | 15 | 38.20 | 23.16 | 0.361 |
| cPIQ | 26 | 32.85 | 21.33 | 15 | 43.73 | 23.11 | 0.135 |
| cFSIQ | 26 | 19.81 | 26.13 | 15 | 32.33 | 25.92 | 0.146 |
| **Intellectual Functioning (≤ 12 years)** | | | | | | | |
| cVIQ | 19 | 34.21 | 25.11 | 13 | 42.31 | 21.68 | 0.352 |
| cPIQ | 19 | 37.00 | 20.46 | 13 | 47.69 | 21.01 | 0.161 |
| cFSIQ | 19 | 25.68 | 25.35 | 13 | 37.15 | 25.35 | 0.205 |

Independent samples t-test was used to conduct analysis

**Table S3**: Relationship between intellectual functioning (standard) scores and *FMR1* mRNA in males and females.

|  | **Males** | |  | **Females** | |  |
| --- | --- | --- | --- | --- | --- | --- |
|  | *n* | *β ± se* | *p* | *n* | *β ± se* | *p* |
| VIQ | 47 | 6.76 *±* 2.75 | **0.018*** | 27 | 9.27 *±* 6.48 | 0.165 |
| PIQ | 51 | 7.13 *±* 1.73 | **<0.001*** | 28 | 3.80 *±* 5.35 | 0.484 |
| FSIQ | 39 | 8.04 *±* 0.73 | **<0.001*** | 27 | 4.09 *±* 6.21 | 0.516 |

Robust regression was used for males while ordinary regression was used for females.

*All p-values < 0.05 after adjustment for multiple testing.

**Table S4**: Relationship between intellectual functioning scores (corrected) and *FMR1* mRNA in males and females with WISC-III (Chile) and MSEL removed

|  | **Males** | |  | **Females** | |  |
| --- | --- | --- | --- | --- | --- | --- |
|  | *n* | *β ± se* | *p* | *n* | *β ± se* | *p* |
| cVIQ | 41 | 8.34 *±* 2.54 | **0.003*** | 21 | 7.82 *±* 7.65 | 0.320 |
| cPIQ | 41 | 8.21 *±* 3.98 | **0.046*** | 21 | 7.07 *±* 7.02 | 0.327 |
| cFSIQ | 41 | 8.55 *±* 3.35 | **0.015*** | 21 | 3.81 *±* 7.82 | 0.632 |

Semi-parametric regression was used for males, adjusted for country, age and ADOS CSS, while robust regression was used for females, adjusted only for ADOS CSS. *p-values < 0.05 after adjustment for multiple testing.

**Table S5**: Relationship between intellectual functioning (standard) scores with *FMR1* mRNA in FM-only with incomplete *FMR1* mRNA silencing and PM/FM mosaic males.

|  | **FM-only with incomplete *FMR1* mRNA silencing** | |  | **PM/FM Mosaic** | |  |
| --- | --- | --- | --- | --- | --- | --- |
|  | *n* | *β ± se* | *p* | *n* | *β ± se* | *p* |
| VIQ | 25 | 19.9 *±* 8.02 | **0.013*** | 10 | 9.26 *±* 3.37 | **0.006*** |
| PIQ | 24 | 12.5 *±* 3.12 | **<0.001*** | 11 | 11.8 *±* 2.77 | **<0.001*** |
| FSIQ | 19 | 11.4 *±* 1.31 | **<0.001*** | 9 | 9.06 *±* 2.61 | **0.001*** |

Robust regression was used to conduct analysis, without adjustment for any covariate.

*p-values < 0.05 after adjustment for multiple testing.
